# The 10^th^ anniversary of patient safety in surgery

**DOI:** 10.1186/s13037-017-0145-x

**Published:** 2017-12-14

**Authors:** Philip F. Stahel, Wade R. Smith, Ernest E. Moore, Philip S. Mehler, Sebastian Weckbach, Fernando J. Kim, Nathan Butler, Hans-Christoph Pape, Ted J. Clarke, Martin A. Makary, Pierre-Alain Clavien

**Affiliations:** 1 Orthopaedics and Neurosurgery, Rocky Vista University, College of Osteopathic Medicine, Parker, CO 80134 USA; 2 Mountain Orthopedic Trauma Surgeons (MOTUS), Swedish Medical Center, Englewood, CO 80113 USA; 3 Department of Surgery, University of Colorado School of Medicine, Denver, CO 80045 USA; 4 Department of Medicine, University of Colorado School of Medicine, Aurora, CO 80045 USA; 5 Department of Orthopedics, University of Ulm, D-89081 Ulm, Germany; 6 West Virginia School of Osteopathic Medicine, Lewisburg, WV 24901 USA; 7 Department of Trauma Surgery, University Hospital Zurich, CH-8091 Zurich, Switzerland; 8 Colorado Physician Insurance Companies, Denver, CO 80320 USA; 9 Department of Surgery, Johns Hopkins School of Medicine, Baltimore, MD 21287 USA; 10 Department of Surgery and Transplantation, University Hospital Zurich, CH-8091 Zurich, Switzerland

**Keywords:** Patient safety in surgery, Journal, Publication metrics, Medical errors, Disclosure, Reporting

This year marks the 10^th^ anniversary of our open-access peer-reviewed journal *“Patient Safety in Surgery”* (PSS). In our launch editorial on November 7, 2007, we made a statement which appears to be still applicable a decade after the journal’s inception: “*Despite the wide range of more than 200 official journals in the field of surgery, there is currently no single medical journal available which specializes on the issue of patient safety in surgery.”* [1]. This notion reflects an apparent void in covering the pertinent topic of surgical patient safety in the scientific literature, and PSS seems to continuously represent a “niche” forefront pilot journal based on its exclusive focus dedicated to surgical patient safety. The journal’s international visibility has continuously grown in the past decade. Current statistics show a global readership of PSS in 185 countries with an average of 230,000 article accesses to the journal’s website each month (Fig. 1). This is an impressive increase compared to historical metrics of just 2000 monthly accesses at the time of the journal’s founding in 2007, and an average of 16,000 accesses per month at the 5-year anniversary mark in 2012 [2].

**Fig. 1:**
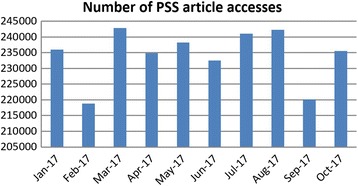
Monthly number of articles accessed on the journal’s website (www.pssjournal.com) in 2017 (January–October). These metrics do not include article downloads from other databases, e.g. PubMed Central

In the past decade, the journal published a total of 325 open-access articles, all of which are cited in PubMed, SpringerLink (https://link.springer.com/journal/13037), and other indexing services that are listed on the journal’s website (www.pssjournal.com). The journal’s overall rejection rate is 20%. The prevalent countries of origin for articles submitted and published in 2017 are depicted in Fig. 2. The highest-ranking publication in the journal of all times is a review article from 2010 entitled *“Complications in colorectal surgery: risk factors and preventive strategies”* [3]. Until present, this particular paper alone has been accessed on the journal’s website more than 110,000 times, and was cited 80 times in other publications [3]. The top-10 most accessed and most cited articles in the journal are listed in Table 1 and Table 2, respectively.

**Fig. 2:**
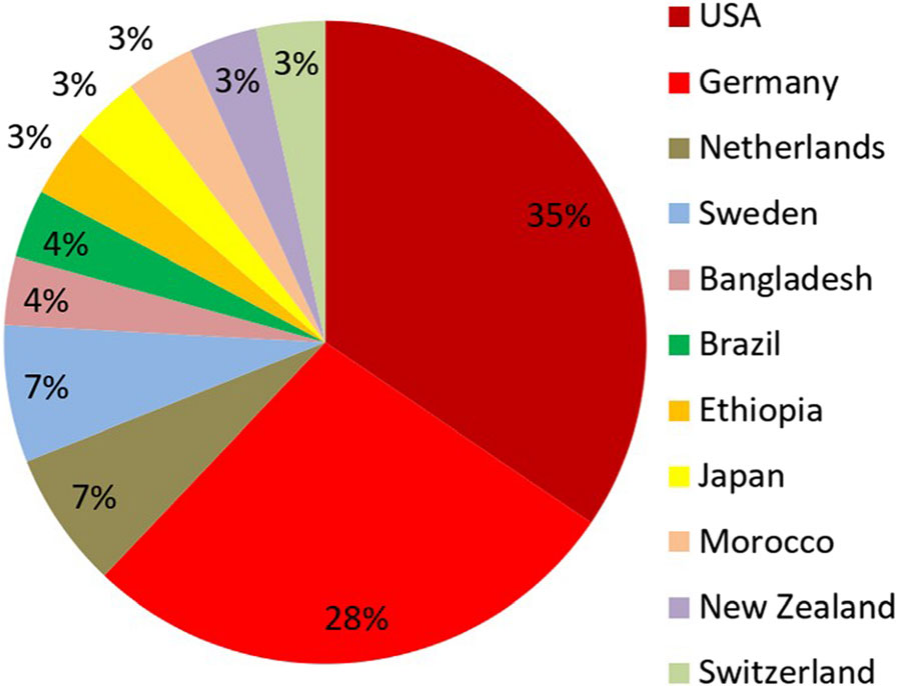
Countries of origin for submitted articles and publications in PSS (YTD 2017)

**Table 1:** Top-10 most accessed articles on the PSS website (as of Nov. 15, 2017)

Rank	Article	Publication year	Accesses (n)
1	Kirchhoff P, *et al*. Complications in colorectal surgery: risk factors and preventive strategies. *Patient Saf. Surg.* 2010, 4:5	2010	114,201
2	Stahel PF, *et al*. Why do surgeons continue to perform unnecessary surgery? *Patient Saf. Surg.* 2017, 11:1	2017	100,785
3	Stornelli N, *et al*. The dangers of lithotomy positioning in the operating room: case report of bilateral lower extremity compartment syndrome after a 90-min surgical procedure. *Patient Saf. Surg.* 2016, 10:18	2016	93,699
4	Subramanian V, *et al*. The risk of intra-urethral Foley catheter balloon inflation in spinal cord-injured patients: Lessons learned from a retrospective case series. *Patient Saf. Surg.* 2016, 10:14	2016	90,381
5	Oldhafer F, *et al*. Monitoring of liver function in a 73-year old patient undergoing ‘Associating Liver Partition and Portal vein ligation for Staged hepatectomy’: case report applying the novel liver maximum function capacity test. *Patient Saf. Surg.* 2016, 10:16	2016	87,966
6	Ten Hagen A, *et al*. Anaphylactic shock during cement implantation of a total hip arthroplasty in a patient with underlying mastocytosis: case report of a rare intraoperative complication. *Patient Saf. Surg.* 2016, 10:25	2016	87,958
7	Weckbach S, *et al*. A survey on patients’ knowledge and expectations during informed consent for spinal surgery: can we improve the shared decision-making process? *Patient Saf. Surg.* 2016, 10:15	2016	87,681
8	Almahmoud K, *et al*. Trends in intubation rates and durations in ventilated severely injured trauma patients: an analysis from the TraumaRegister DGU®. *Patient Saf. Surg.* 2016, 10:24	2016	85,979
9	Schmitt JW, *et al*. Is total hip arthroplasty safely performed in lung transplant patients? Current experience from a retrospective study of the Zurich lung transplant cohort. *Patient Saf. Surg.* 2016, 10:17	2016	84,986
10	Stahel PF, *et al*. Introducing the “Bone-Screw-Fastener” for improved screw fixation in orthopedic surgery: a revolutionary paradigm shift? *Patient Saf. Surg.* 2017, 11:6	2017	82,464

These metrics exclude access numbers through PubMed and other indexing databases

**Table 2:** Top-10 most cited PSS articles in other publications (as of Nov. 15, 2017)

Rank	Article	Publication year	Citations (n)
1	Kirchhoff P, *et al*. Complications in colorectal surgery: risk factors and preventive strategies. *Patient Saf. Surg.* 2010, 4:5	2010	80
2	de Vries EN, *et al*. The SURgical PAtient Safety System (SURPASS) checklist optimizes timing of antibiotic prophylaxis. *Patient Saf. Surg.* 2010, 4:6	2010	52
3	Lynn LA, Curry JP. Patterns of unexpected in-hospital deaths: a root cause analysis. *Patient Saf. Surg.* 2011, 5:3	2011	49
4	Zegers M, *et al*. The incidence, root-causes, and outcomes of adverse events in surgical units: implication for potential prevention strategies. *Patient Saf. Surg.* 2011, 5:13	2011	36
5	O'Connor P, *et al*. Surgical checklists: the human factor. *Patient Saf. Surg.* 2013, 7:14	2013	28
6	Liu SS, *et al*. Risk of postoperative hypoxemia in ambulatory orthopedic surgery patients with diagnosis of obstructive sleep apnea: a retrospective observational study. *Patient Saf. Surg.* 2010, 4:9	2010	21
7	Youngson GG, Flin R. Patient safety in surgery: non-technical aspects of safe surgical performance. *Patient Saf. Surg.* 2010, 4:4	2010	20
8	Unbeck M, *et al*. Is detection of adverse events affected by record review methodology? An evaluation of the *Harvard Medical Practice Study* method and the *Global Trigger Tool*. *Patient Saf. Surg.* 2013, 7:10	2013	18
9	Andersson AE, *et al*. The application of evidence-based measures to reduce surgical site infections during orthopedic surgery - report of a single-center experience in Sweden. *Patient Saf. Surg.* 2012, 6:11	2012	18
10	Robinson Y, *et al*. Increased occurrence of spinal fractures related to ankylosing spondylitis: a prospective 22-year cohort study in 17,764 patients from a national registry in Sweden. *Patient Saf. Surg.* 2013, 7:2	2013	17

Another important aspect related to the journal’s global visibility is reflected by the so-called “Altmetric Attention Score”. This represents an emerging tool designed to assess the public attention that scholarly articles receive through the media, news outlets, blogs, and social media. The score is influenced by the quantity of posts that mention an article and by the quality of the source of posting. Of note, Altmetric measures public attention, not scientific quality. The PSS article with the highest Altmetric Attention Score of all times was a 2017 editorial entitled *“Why do surgeons continue to perform unnecessary surgery?”* [4]. Impressively, the article’s score of 414 falls into the top-5% of all research outputs scored by Altmetric [4]. These statistics reflect on an increased international visibility of the journal in the public media and other outlets. A list of the top-10 PSS articles ranked by Altmetric scores is depicted in Table 3.

**Table 3:** Top-10 Altmetric Attention Scores (as of Nov. 15, 2017)

Rank	Article	Publication year	Altmetric score
1	Stahel PF, *et al*. Why do surgeons continue to perform unnecessary surgery? *Patient Saf. Surg.* 2017, 11:1	2017	414
2	Caesar U, *et al*. Incidence and root causes of cancellations for elective orthopaedic procedures: a single center experience of 17,625 consecutive cases. *Patient Saf. Surg.* 2014, 8:24	2014	50
3	Page AE. Safety in surgery: the role of shared decision-making. *Patient Saf. Surg.* 2015, 9:24	2015	31
3	Lynn LA. The diagnosis of sepsis revisited - a challenge for young medical scientists in the twenty-first century. *Patient Saf. Surg.* 2014, 8:1	2014	31
5	Nwosu A. The horror of wrong-site surgery continues: report of two cases in a regional trauma centre in Nigeria. *Patient Saf. Surg.* 2015, 9:6	2015	25
5	Zeeshan MF, *et al*. Incidence of adverse events in an integrated US healthcare system: a retrospective observational study of 82,784 surgical hospitalizations. *Patient Saf. Surg.* 2014, 8:23	2014	25
7	Pfeifer R, Pape HC. Missed injuries in trauma patients: A literature review. *Patient Saf. Surg.* 2008, 2:20	2008	22
8	Iglar PJ, Hogan KJ. Vitamin D status and surgical outcomes: a systematic review*.* *Patient Saf. Surg.* 2015, 9:14	2015	21
9	Charles R, *et al*. How to perform a root cause analysis for workup and future prevention of medical errors: a review. *Patient Saf. Surg.* 2016, 10:20	2016	19
10	Curry JP, Jungquist CR. A critical assessment of monitoring practices, patient deterioration, and alarm fatigue on inpatient wards: a review. *Patient Saf. Surg.* 2014, 8:29	2014	18

In spite of these impressive metrics, which are reflective of a successful development and increased visibility of the journal since its inception in 2007, there is much work to be done. Notwithstanding significant developments in medical care and technology, the “modern age” of patient safety in the twenty-first century continues to fall short of protecting patients from unnecessary treatment, preventable harm, and death. Shockingly, current statistics have identified medical errors as the 3rd leading cause of death in the United States, secondary only to cardiovascular disease and cancer [5]. The hidden epidemic is subtantiated by evidence-based estimates of more than 400,000 preventable annual deaths occurring in United States hospitals every year [6], which does not take into account the innumerable preventable deaths subsequent to unsafe hospital discharges and medication errors in the outpatient setting. In spite of this egregious system failure, the medical profession continues to accept errors that lead to preventable patient harm as an unfortunate and inevitable “side effect” of modern health care. The unintentional void created by the absence of physician leadership in the field of patient safety has meanwhile been filled by other stakeholders, including patient advocacy groups, malpractice lawyers, and legislators. It is time for a change in mindset. A famous Chinese proverb fittingly states that “the best time to plant a tree was forty years ago; the second-best time is now.” Today is the time for the medical profession to make up for past negligence by taking the lead in driving patient safety as an irrefutable responsibility. The mission of this journal is to continue to provide an international forum for reporting, discussing, and mitigating errors and failures that lead to preventable patient harm and adverse outcomes. We would like to thank our readers, authors, and reviewers for their trust and loyalty over the past decade, and we hope to count on your continuing support of the journal’s mission.

## References

[1] Stahel PF, Clavien PA, Hahnloser D, Smith WR (2007). A new journal devoted to patient safety in surgery: the time is now!. Patient Saf Surg.

[2] Stahel PF, Smith WR, Hahnloser D, Nigri G, Mauffrey C, Clavien PA (2012). The 5th anniversary of patient safety in surgery - from the Journal's origin to its future vision. Patient Saf Surg.

[3] Kirchhoff P, Clavien PA, Hahnloser D (2010). Complications in colorectal surgery: risk factors and preventive strategies. Patient Saf Surg.

[4] Stahel PF, VanderHeiden TF, Kim FJ (2017). Why do surgeons continue to perform unnecessary surgery?. Patient Saf Surg.

[5] Makary MA, Daniel M (2016). Medical error - the third leading cause of death in the US. BMJ.

[6] James JT (2013). A new, evidence-based estimate of patient harms associated with hospital care. J Patient Saf.

